# Letter to the Editor for the article ‘Comparative dose-response study on the infusion of norepinephrine combined with intravenous ondansetron versus placebo for preventing hypotension during spinal anesthesia for cesarean section: a randomised controlled trial’

**DOI:** 10.1097/JS9.0000000000001571

**Published:** 2024-05-15

**Authors:** Jiaan Lu, Hao Chi, Guanhu Yang, Qibiao Wu

**Affiliations:** a Faculty of Chinese Medicine, State Key Laboratoryof Quality Research in Chinese Medicine,and University Hospital, Macau University of Science and Technology, Macau, Macao SAR, People’s Republic of China; b Clinical Medical College, Southwest Medical University, Luzhou, China; c Department of Specialty Medicine, Ohio University, Athens, Ohio, USA

*Dear Editor,*

I am writing to express my gratitude for the insightful research conducted by Sheng *et al*.^1^ titled ‘Comparative dose-response study on the infusion of norepinephrine combined with intravenous ondansetron versus placebo for preventing hypotension during spinal anesthesia for cesarean section: a randomized controlled trial.’ The authors have delved into a clinically relevant area and addressed the demand for improved strategies to prevent hypotension during cesarean sections under spinal anesthesia.

The study’s methodological rigor is evident in its randomized controlled design and adherence to ethical standards, which deserves recognition. The inclusion of a diverse group of parturients and meticulous documentation of demographic and anesthesia-related data make the findings more reliable and applicable. Additionally, the use of probit regression analysis to explore the dose–response relationship of prophylactic norepinephrine infusion adds depth to the investigation and also facilitates a nuanced understanding of its effectiveness.

The results emphasize the potential of intravenous ondansetron in reducing the dose requirement of prophylactic norepinephrine infusion and offer a promising avenue for improving perioperative care in parturients undergoing elective cesarean sections. This finding holds significant implications for clinical practice, suggesting refinements to current protocols to minimize hypotension and mitigate potential adverse effects associated with excessive vasopressor use.

However, despite the study’s strengths, several limitations should be noted.

Firstly, while the sample size calculation was meticulous, the exclusion of 18 parturients could introduce bias and affect result generalizability^1^. Additionally, while side effects were reported, further exploration of confounding factors influencing their occurrence could enrich the discussion and strengthen the study’s integrity^1^.

Moreover, the absence of significant differences in neonatal outcomes between the intervention and control groups is noteworthy. However, a more extensive evaluation which includes long-term follow-up data, could offer valuable insights into the intervention’s impact on neonatal health beyond the immediate postoperative period^1^.

Furthermore, I offer some suggestions for enhancing the study: Table 1.

**Table 1:** The suggestions aim to refine the study further and enhance its contribution to the academic community.

Number	Consideration	Description	Reference
1	Methodological considerations	Consider employing a double-blind design to minimize subjective bias and ensure standardization during intervention implementation for consistency and comparability.	^2,3^
2	Safety considerations	Besides discussing intervention effectiveness, the study should thoroughly assess potential safety issues, especially regarding pregnant women and fetuses.	^1^
3	Result interpretation	Ensure the article provides a comprehensive explanation of results, considering potential influencing factors when discussing differences between intervention and control groups.	^1^
4	Future research directions	Besides highlighting current study limitations, the study should explore future research directions, such as the necessity of longer-term follow-up to assess intervention effects.	^4,5^
5	Article contributions	Apart from summarizing the main findings, reflect on the overall significance for clinical practice and research, suggesting changes and improvements for future studies and practice.	^1^

In summary, Sheng *et al*.’s study makes valuable contributions to obstetric anesthesia literature by elucidating the dose–response relationship of prophylactic norepinephrine infusion and intravenous ondansetron, paving the way for safer perioperative management strategies for cesarean section patients. Addressing identified limitations can strengthen the effectiveness and applicability of results, fostering advancements in obstetric anesthesia practice.

Finally, we truly thank the authors for their excellent and important work.

## Ethical approval

Not applicable.

## Consent

Not applicable.

## Sources of funding

This work was supported by the Science and Technology Development Fund, Macau SAR (Nos.: 0098/2021/A2 and 0048/2023/AFJ), the National Natural Science Foundation of China (No.: 82361168663), and Macau University of Science and Technology’s Faculty Research Grant (No: FRG-23-003-FC).

## Author contribution

J.L., G.Y., H.C., and Q.W.: conceptualization, methodology, validation, formal analysis, investigation, resources, data curation, writing – original draft, writing – review and editing, supervision, and project administration.

## Conflicts of interest disclosure

The authors declares no conflicts of interest.

## Research registration unique identifying number (UIN)

Not applicable.

## Guarantor

Jiaan Lu, Guanhu Yang, Hao Chi, and Qibiao Wu.

## Data availability statement

Not applicable.

## Provenance and peer review

Not applicable.
